# The Importance of the Stem Cell Marker Prominin-1/CD133 in the Uptake of Transferrin and in Iron Metabolism in Human Colon Cancer Caco-2 Cells

**DOI:** 10.1371/journal.pone.0025515

**Published:** 2011-09-26

**Authors:** Erika Bourseau-Guilmain, Audrey Griveau, Jean-Pierre Benoit, Emmanuel Garcion

**Affiliations:** Laboratoire d'Ingénierie de la Vectorisation Particulaire, Inserm, UMR-S 646, Université d'Angers, Angers, France; Vanderbilt University Medical Center, United States of America

## Abstract

As the pentaspan stem cell marker CD133 was shown to bind cholesterol and to localize in plasma membrane protrusions, we investigated a possible function for CD133 in endocytosis. Using the CD133 siRNA knockdown strategy and non-differentiated human colon cancer Caco-2 cells that constitutively over-expressed CD133, we provide for the first time direct evidence for a role of CD133 in the intracellular accumulation of fluorescently labeled extracellular compounds. Assessed using AC133 monoclonal antibody, CD133 knockdown was shown to improve Alexa488-transferrin (Tf) uptake in Caco-2 cells but had no impact on FITC-dextran or FITC-cholera-toxin. Absence of effect of the CD133 knockdown on Tf recycling established a role for CD133 in inhibiting Tf endocytosis rather than in stimulating Tf exocytosis. Use of previously identified inhibitors of known endocytic pathways and the positive impact of CD133 knockdown on cellular uptake of clathrin-endocytosed synthetic lipid nanocapsules supported that CD133 impact on endocytosis was primarily ascribed to the clathrin pathway. Also, cholesterol extraction with methyl-β-cyclodextrine up regulated Tf uptake at greater intensity in the CD133^high^ situation than in the CD133^low^ situation, thus suggesting a role for cholesterol in the inhibitory effect of CD133 on endocytosis. Interestingly, cell treatment with the AC133 antibody down regulated Tf uptake, thus demonstrating that direct extracellular binding to CD133 could affect endocytosis. Moreover, flow cytometry and confocal microscopy established that down regulation of CD133 improved the accessibility to the TfR from the extracellular space, providing a mechanism by which CD133 inhibited Tf uptake. As Tf is involved in supplying iron to the cell, effects of iron supplementation and deprivation on CD133/AC133 expression were investigated. Both demonstrated a dose-dependent down regulation here discussed to the light of transcriptional and post-transciptional effects. Taken together, these data extend our knowledge of the function of CD133 and underline the interest of further exploring the CD133-Tf-iron network.

## Introduction

Following the use of new monoclonal antibodies raised against neuroepithelial and hematopoietic stem cells, CD133, also known in humans and rodents as Prominin-1, was first isolated and cloned in 1997 [1], [2], [3]. CD133 is a five-domain transmembrane protein, composed of an N-terminal extracellular tail, two small cytoplasmic loops, two large extracellular loops containing seven potential glycosylation sites and a short C-terminal intracellular tail that can be alternatively spliced [4] or phosphorylated [5].

Despite constant research efforts, the biological function of CD133 remains largely unknown. Among notorious phenotypes, it has been shown that a truncated CD133, which is not transported to cell membrane, leads to human retinal degeneration [6]. Underlining this important observation, analysis of a generation of CD133-deficient mice revealed that, while expressed very early during retinal development, CD133 acted as a key regulator of disk morphogenesis and that loss of CD133 caused photoreceptor degeneration and blindness [7]. In addition, AC133, a glycosylated epitope of CD133 protein initially associated with embryonic stem cells [8] and a variety of somatic stem cells, was extensively described as a putative cancer stem cell marker in blood, brain, colon, prostate, lung, breast, liver, and skin cancers [9], [10]. Other investigations revealed that CD133 is linked to cell metabolism as a glucose responsive gene in myotubes [11], as well as providing evidence for bioenergetic stress [12] and of non-exposure to high oxygen tension in gliomas (Bourseau-Guilmain et al., submitted).

At the subcellular level, CD133 is preferentially localized in plasma membrane protrusions and microvilli [13]. From there, CD133 can bind to cholesterol [14] and interact with gangliosides [15]. As membrane protrusions and microvilli enable extension of the membrane surface in order to increase cell exposure to the extracellular space, these observations provide important clues to identifying the molecular role of CD133, notably by considering cellular exchanges with the microenvironment. Indeed, CD133 was found in membrane vesicles distinct from exosomes that were released from epithelial cells during differentiation [16].

In parallel to these outside-in signals, cholesterol and sphingolipids segregate in lipid raft membrane microdomains implicated in inside-out signaling and endocytosis [17], [18]. Considering the tight relation between CD133 and cholesterol, plus its possible link to sphingolipids and exposure to the extracellular space, we hypothesized that CD133 is involved in endocytosis: a fundamental process by which extracellular compounds are internalized and distributed to intracellular compartments.

In the present study, using the RNA-interference strategy and undifferentiated human colon cancer Caco-2 cells that constitutively over-expressed CD133/AC133, we provide for the first time evidence for a role of CD133 in the intracellular accumulation of extracellular compounds, notably exemplified by transferrin (Tf). In addition to data that establish a role for CD133 in endocytosis, we also demonstrate that CD133 itself is regulated by iron, thus supporting the existence of a Tf-CD133-iron network. These new observations are discussed in the light of the CD133 pattern of expression and current knowledge in the field.

## Materials and Methods

### Cell culture

Undifferentiated human colon carcinoma Caco-2 cells (American type culture collection: HTB-37™) were cultured at 37°C in an atmosphere of 5% CO_2_ in Dulbecco's Modified Eagle Medium (DMEM; Lonza, Levallois-Perret, France) containing 4.5 g/L glucose and L-glutamine. The medium was added with 10% of fetal bovine serum (FBS; Lonza, Verviers, Belgium), 1% antibiotics (10 units/mL penicillin, 10 mg/mL streptomycin, 25 µg/mL amphotericin B; Sigma-Aldrich, Saint-Louis, USA, MO) and 1% of non-essential amino acids (NEAA; Lonza, Verviers, Belgium). When cells reached 80% confluence, they were dissociated in 0.5% porcine trypsin and 0.2 g/mL EDTA (Lonza, Verviers, Belgium) before re-plating on uncoated plastic flasks at 15×10^3^ cells/cm^2^. Half the medium was replaced by fresh medium every two days.

### siRNA knockdown of CD133

Caco-2 cells were plated in six-well plates with 2.3×10^5^ cells per well in 2 mL of the aforementioned medium for 24 h. Medium was removed and cells were transfected with 30 nM of RNA oligonucleotide duplexes (Sigma-Aldrich) using the N-TER reagent according to manufacturer's instructions (Sigma-Aldrich). N-TER/siRNA complexes were then incubated in Caco-2 medium for 48 h at 37°C in an atmosphere containing 5% CO_2_. siRNA were then removed and replaced by fresh medium for 24 h. A sequence scramble was used as negative control: 5′-GUCCGGAAUUACCAUGAGUdTdT-3′. The sequence used to perform targeted RNA interference inhibition of CD133 was 5′-CCCUUAAUGAUAUACCUGAdTdT-3′.

### AC133 immunolabeling

Caco-2 cells exposed to siRNA were collected and dissociated using Versene (Lonza). Cells were incubated with 5 µg/mL AC133 antibody (Miltenyi Biotech, Paris, France) or IgG1κ isotype control (BD-Biosciences, Le Pont-de-Claix, France) for 1 h at 4°C in PBS containing 5% FBS and 0.02% sodium azide. Cells were then washed three times in PBS containing 5% FBS and 0.02% sodium azide, and incubated for 30 minutes at 4°C with FITC-conjugated goat anti-mouse IgG F(ab')2 fragment polyclonal antibody (Dakocytomation, Trappes, France) at 20 µg/mL in PBS containing 5% FBS and 0.02% sodium azide. Following three more washes in PBS containing 5% FBS and 0.02% sodium azide, cells were re-suspended in PBS containing 2% formaldehyde and 0.02% sodium azide.

### Flow cytometry

A BD FACSCalibur™ fluorescent-activated flow cytometer and the BD CellQuest™ software (BD-Biosciences) were used for flow cytometry acquisition. Analysis was carried out using WinMDI 2.9 software (Scripps Institute, La Jolla, CA, USA).

### Synthesis of Nile red-labeled lipid nanocapsules (NR-LNC)

50 nm Nile red (NR)-labeled lipid nanocapsules (LNC) (NR-LNC) that incorporated the fluorescent compound NR were prepared as previously described [19] by using a phase inversion process that follows the formation of an oil/water microemulsion containing triglycerides (Labrafac® WL 1349, Gattefossé, Saint-Priest, France), a non ionic hydrophilic surfactant (Solutol® HS 15, Gmbh, Ludwigshafen, Germany) and lecithins (Lipoïd® S75-3, Gmbh). NR was dissolved in acetone at 1% (w/w), and the resulting NR solution was incorporated in Labrafac® at 1∶10 (w/w). Thus, 846 mg Solutol®, 75 mg Lipoïd®, 1029 mg Labrafac® containing NR, 89 mg NaCl and 2975 mg water were mixed and heated under magnetic stirring up to 85°C. Three cycles of progressive heating and cooling in between 85°C and 60°C were then realized. They were finally followed by an irreversible shock induced by dilution with 12.5 mL of 0°C deionised water added into the inversion phase zone. Size exclusion and high-pressure liquid chromatography (HPLC) assays demonstrated a complete encapsulation and retention of NR as previously observed [19]. LNC were analysed for their size distribution using a Malvern Zetasizer® Nano Series DTS 1060 (Malvern Instruments S.A., Worcestershire, UK).

### Internalization of transferrin (Tf), dextran (Dx), cholera toxin subunit B (CTB) and lipid nanocapsules (LNC)

The medium of transfected cells was removed and replaced by DMEM supplemented with 1% of serum-free N1 medium (Sigma Aldrich) for 1 h. The three following compounds, Tf-Alexa 488 at 0.5 and 5 µg/mL (Invitrogen, Cergy Pontoise, France), Dx-FITC at 0.5 and 5 mg/mL (Sigma Aldrich) and CTB-FITC at 1 and 10 µg/mL (Invitrogen), or instead, NR-LNC at 1/1000 dilution from initial suspension thus corresponding to a 100 µg/mL concentration, were then added to the medium for 1 h. Cells were then dissociated using Versene (Lonza). To enable determination of the fraction of labeled molecules or particles that was effectively internalized within cells, extracellular fluorescence was quenched using 0.4% trypan blue (Sigma Aldrich). Cells were then washed in PBS and re-suspended in PBS containing 2% formaldehyde and 0.02% sodium azide. Internalization of fluorescently labeled Tf, Dx, CTB and LNC was monitored by flow cytometry.

### Analysis of the release of Tf from Caco-2 cells after internalization

Control and CD133-specific siRNA were used to generate CD133^high^ and CD133^low^ transfected Caco-2 cells, respectively. Cells were then incubated with Tf-Alexa 488 for 2 h at 37°C/5% CO_2_ before the extracellular media was removed, washed and replaced by fresh medium free from Tf-Alexa 488. After further incubation at 37°C/5% CO_2_ for 1 h, 2 h and 3 h, the remaining intracellular Tf-Alexa 488 fluorescence, representing the amount of Tf-Alexa 488 that was not recycled to the extracellular compartment, was measured by semi-quantitative flow cytometry as described above.

### Cell treatment with chemical inhibitors of endocytosis and cellular uptake of Tf

Previously identified chemical inhibitors of known endocytic pathways were used as previously described [19], [20], [21]. Briefly, transfected Caco-2 cells were pre-treated with inhibitors for 1 h at 37°C in N1 medium. Chlorpromazine (10 µg/mL; Sigma-Aldrich) was used to inhibit clathrin-mediated transport [22], while filipin (1 µg/L; Sigma-Aldrich) and dimethylamiloride (DMA, 10 µM; Sigma-Aldrich) were used to inhibit caveolae-dependent endocytosis [23] and macropinocytosis [24], respectively. Cholesterol depletion was obtained by a 2 h pretreatment with methyl-β-cyclodextrine (MβCD, 10 mM; Sigma-Aldrich) in the presence of lovastatin (1 µg/mL; Sigma-Aldrich) [25], [26]. Subsequently, cellular uptake of 5 µg/mL Tf-Alexa 488 was monitored by flow cytometry analysis as described above. For cholesterol inhibition, 1 µg/mL lovastatin was also maintained in the medium during the treatment with Tf-Alexa 488.

### Analysis of Tf uptake by Caco-2 cells after treatment with the AC133 antibody

Caco-2 cells were plated at 2.3×10^5^ in 24-well plates in 400 µL of serum containing medium. After a 24 h initial incubation at 37°C/5% CO_2_ the initial medium was replaced by N1 serum free medium before incubation with either 0, 5, or 10 µg/mL of the AC133 antibody or of isotype control IgG1κ. Tf-Alexa 488 was then added at 5 µg/mL and incubated at 37°C/5% CO_2_ for 1 h to study the impact of the immunoglobulin treatment on Tf-Alexa 488 uptake. Tf-Alexa 488 uptake was quantified by flow cytometry as described above.

### Antibody recognition of the Tf receptor at surface the Caco-2 cell depending on the level of CD133 expression

Caco-2 cells exposed to CD133 or control siRNA were collected and dissociated using Versene (Lonza). Cells were incubated with 5 µg/mL CD71 mouse monoclonal antibody that recognize the Tf receptor (TfR or CD71 antigen) (clone M-A712, BD-Biosciences) or with 5 µg/mL IgG2a, κ isotype control (BD-Biosciences) to proceed for immunolabeling and flow cytometry as described above for AC133 cell surface recognition.

### Immunocytochemisty combined with confocal laser scanning microscopy for the identification of AC133, CD71 and clathrin heavy chain within Caco-2 cells

Caco-2 cells were plated at 4×10^3^ cells per well in eight-well Lab-Tek Chamber Slides (Nunc, Roskilde, Denmark) in 300 µL DMEM containing 10% FCS for 24 h. They were then exposed to CD133 or control siRNA as described above before to proceed to immunocytochemistry. Cells were then washed with PBS and fixed with 4% paraformaldehyde in PBS (pH 7.4) for 20 minutes at 4°C. After washes in PBS, cells were exposed for 60 minutes at room temperature to a blocking solution of PBS containing 4% of bovine serum albumin (Sigma-Aldrich) and 10% of normal goat serum (Sigma-Aldrich). Primary monoclonal antibodies against CD133 (IgG1κ, Miltenyi Biotech), TfR or CD71 (IgG2aκ, BD-Biosciences) or clathrin heavy chain (CHC) (IgG1, BD-Biosciences) were incubated at 5 µg/mL in PBS/BSA 4% for overnight at 4°C. Note that for CHC intracellular immunolabeling, Triton® X-100 was added at 0.1% during the blocking procedure for cell permeation. After washes, a secondary horse anti-mouse biotin-conjugated antibody (Vector, Burlingame, CA) was applied at 1/100 in PBS/BSA 4% for 1 h at room temperature. After additional washes, Streptavidin Fluo Probe Alexa 488 (Interchim, Montluçon, France), diluted 1/700, was used. Cells were finally washed and mounted under coverslip in fluorescent mounting medium (Dakocytomation). Confocal microscope images were obtained by using an Olympus Fluoview™ FU 300 confocal laser scanning microscopy imaging system (Paris, France).

### Iron treatments

Caco-2 cells were plated in six-well plates with 2.3×10^5^ cells per well in 2 mL for 24 h. Before iron treatment started, FBS containing medium was replaced by serum free N1 medium for an additional 24 h. Cells were then treated with various concentrations (50–800 µM) of ferric nitrilotriacetic acid (Fe-NTA) for 72 h at 37°C/5% CO_2_, as indicated. Fe-NTA (molar ratio 1∶4) was extemporaneously prepared as a 20-mM stock from NTA and ferric chloride hexahydrate (Sigma-Aldrich). Cells were alternatively treated with FeSO_4_ (Sigma-Aldrich), another iron donor [27], [28], at either 150 or 300 µM for 24 h.

### Iron deprivation and treatment with hypoxia-mimetic agents

Following a similar culture procedure as before iron treatment, cells were instead treated for 24 h with an iron chelator, also known as a hypoxia-mimetic agent, Desferrioxamine (DFO, Sigma-Aldrich) at 100 and 150 µM [29], [30]. Alternatively, they were treated for 24 h with a hypoxia-mimetic agent that works independently from iron deprivation, Cobalt dichloride (CoCl_2_, Sigma-Aldrich) at 100 and 150 µM [31].

### Database, bioinformatics

To search for putative iron responsive element (IRE) sequences within the 3′ and 5′ untranslated region (UTR) of human CD133 mRNA, the sequences used in this study (among which *Homo sapiens* prominin 1 transcript variant 2, NM_001145847.1) were obtained from NCBI GenBank. All sequence alignments were done using the ClustalW computer program from the EMBL European Bioinformatics Institute (Heidelberg, Germany). The SIREs (searching for IREs) web server (http://ccbg.imppc.org/sires/) was also used for prediction of iron responsive elements in RNA [32].

### Statistical analysis

XLSTAT 2006 Version 2006.3 (Addinsoft Paris, France) was used for data analysis. The statistical significance of each experiment was determined by a Dunnett's test. The tests were considered as significant with p values <0.05.

## Results

### Impact of specific siRNA-mediated knockdown of CD133 on the intracellular accumulation of Tf, Dx and CTB in non-differentiated Caco-2 cells

**Figure 1:**
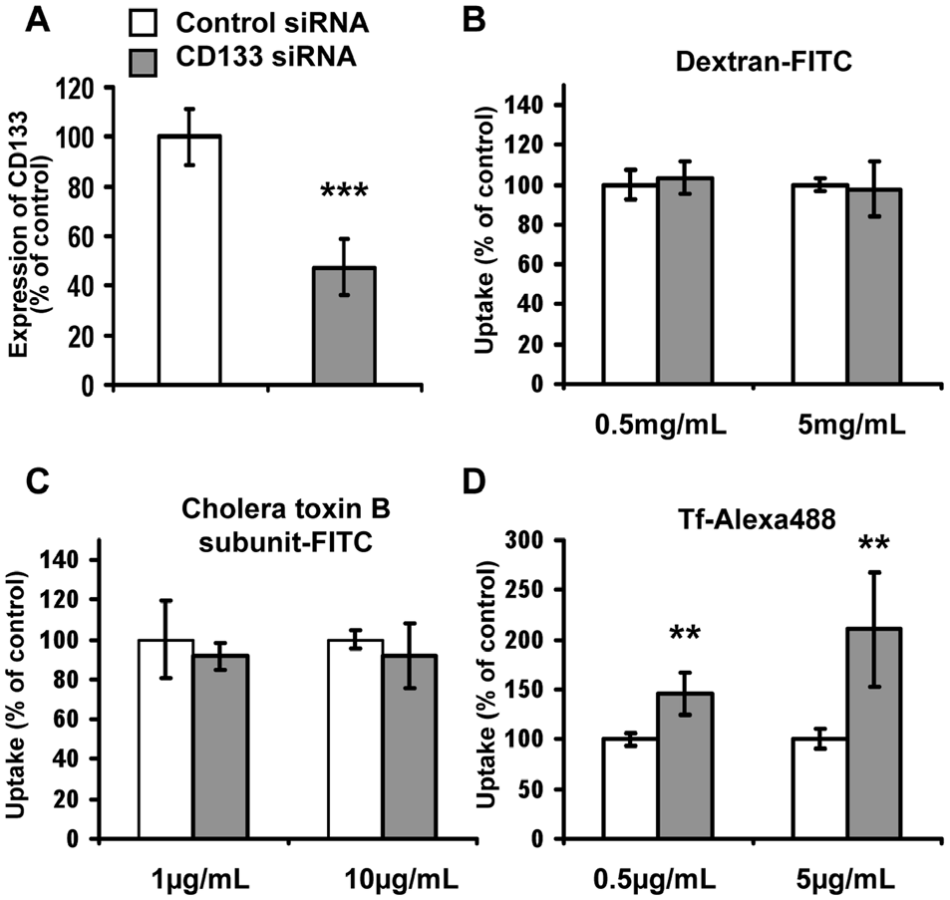
Specific siRNA mediated knockdown of CD133 within non-differentiated Caco-2 cells led to an increase in Tf intracellular accumulation but had no impact on Dx and CTB uptake. A) Immunofluorescence associated flow cytometric analysis of CD133 expression on non-differentiated Caco-2 cells treated with 30 nM of control or CD133-specific siRNA. Results are expressed as a percentage of the control treatment, representing the geomean fluorescence intensity levels obtained after AC133 immunostaining of cells treated with irrelevant siRNA (CD133^high^ Caco-2 cells); note the effective down regulation of CD133 when CD133-specific siRNA (CD133^low^ Caco-2 cells) was used. B–D) Flow cytometric analysis of intracellular uptake of Dx-FITC (B), CTB-FITC (C) and Tf-Alexa 488 (D) within CD133^high^ and CD133^low^ Caco-2 cells after 1 h of incubation at 37°C/5%CO_2_. Results are expressed as percentage of control, thus representing the geomean fluorescence intensity levels obtained for cells treated with vehicle alone. Data represented mean ± s.e.m. obtained from three independent experiments. Dunnett's test: **p<0.01, ***p<0.001

To determine the potential influence of CD133 on the intracellular accumulation of extracellular compounds, non-differentiated human colon carcinoma Caco-2 cells, which naturally express high levels of CD133, were used. Using either siRNA that does not lead to transcriptional down-regulation (control siRNA) or siRNA that does down regulate targeted CD133 mRNA and protein expression (CD133 siRNA), two situations were analyzed: Caco-2 cells expressing high levels of CD133, and Caco-2 cells expressing low levels of CD133. Figure 1A shows that treatment of Caco-2 cells with 30 nM of CD133 siRNA effectively led to a reduction of 52±11% in CD133 protein expression, analyzed by flow cytometry immunofluorescence (AC133 antibody) compared to the control siRNA. To analyze Caco-2 cell pinocytic ability in the CD133^high^ and CD133^low^ situations, the intracellular accumulation of exogenous markers of pinocytic pathways was then monitored. Although alternative internalization pathways have been described depending on cell types and differentiation status (for review see [33]), Tf-Alexa 488, Dx-FITC and CTB-FITC were used as prototype markers of receptor mediated endocytosis [34], fluid phase endocytosis [24], [35] and caveolae dependent endocytosis [23], [36], respectively. Flow cytometry measurement of intracellular fluorescence after 1 h exposure of CD133^high^-Caco-2 cells to either 0.5 or 5 mg/mL Dx-FITC, 1 or 10 µg/mL CTB-FITC and 0.5 or 5 µg/mL Tf-Alexa 488 enabled 100% uptake to be measured compared to the basal geomean fluorescent intensity of vehicle alone treated cells representing 0% uptake. Although CD133 knockdown had no impact on intracellular accumulation of Dx-FITC (Figure 1B) or on CTB-FITC (Figure 1C), cellular uptake of Tf-Alexa 488 was significantly amplified in CD133^low^-Caco-2 cells whatever the concentration tested (Figure 1D). These data thus established for the first time that CD133 act as a modulator of intracellular accumulation of exogenous compounds.

### Effect of siRNA mediated knockdown of CD133 on Tf exocytosis

**Figure 2:**
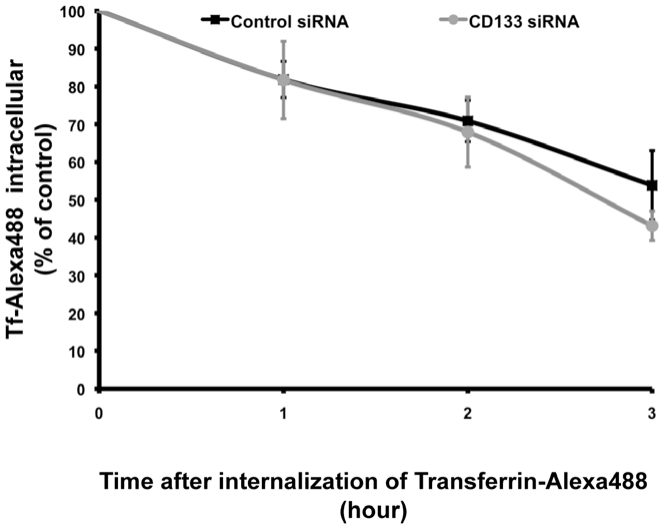
Specific siRNA mediated knockdown of CD133 within non-differentiated Caco-2 cells did not affect short-term Tf exocytosis. CD133^high^ (control siRNA) and CD133^low^ (CD133 siRNA) non-differentiated Caco-2 cells were exposed to Tf-Alexa 488 for 2 h at 37°C/5% CO_2_ before the extracellular medium was removed, washed and replaced by fresh medium free from Tf-Alexa 488. Amounts of Tf-Alexa 488 that were not recycled to the extracellular compartment were measured by flow cytometric analysis after further cell incubation at 37°C/5% CO_2_ for 1 to 3 h. Data are expressed as a % of Tf-Alexa 488 initially internalized. They represent mean ± s.e.m. obtained from three independent experiments.

In view of the fact that CD133 appeared to be an inhibitor of cellular uptake of Tf while having no impact on Dx and CTB, we further focused on the relation between CD133 expression and Tf accumulation. The potential effect of siRNA mediated knockdown of CD133 on Tf-Alexa 488 exocytosis was therefore investigated. For this purpose, CD133^high^ and CD133^low^ non-differentiated Caco-2 cells were incubated with Tf-Alexa 488 for 2 h at 37°C/5% CO_2_ before the extracellular medium was removed, washed and replaced by fresh medium free from Tf-Alexa 488. After further incubation for 1, 2 and 3 h at 37°C/5% CO_2_ the amount of Tf-Alexa 488 that was not recycled to the extracellular compartment was measured by flow cytometry as described in Materials and Methods. Data presented in Figure 2 established that intracellular levels of Tf-Alexa 488 decreased with incubation time. After 1 h of incubation more than 80% of the internalized Tf-Alexa 488 remained within cells in both CD133^high^ and CD133^low^ expressing cells while after a 3 h of incubation, 54±9% and 43±4% of the internalized Tf-Alexa 488 remained within CD133^high^ and CD133^low^ expressing cells, respectively. However at all times studied, no significant difference was observed depending on CD133 expression levels (Figure 2). These observations emphasized that although Tf recycling occurred in both CD133^high^ and CD133^low^ non-differentiated Caco-2 cells, short term differences in intracellular Tf accumulation are due essentially to the impact of CD133 on endocytosis mechanisms rather than exocytosis.

### Effect of chemical inhibitors of known endocytic pathways on the uptake of Tf by non-differentiated Caco-2 cells depending on the level of CD133 expression underlined the importance of the clathrin pathway

**Figure 3:**
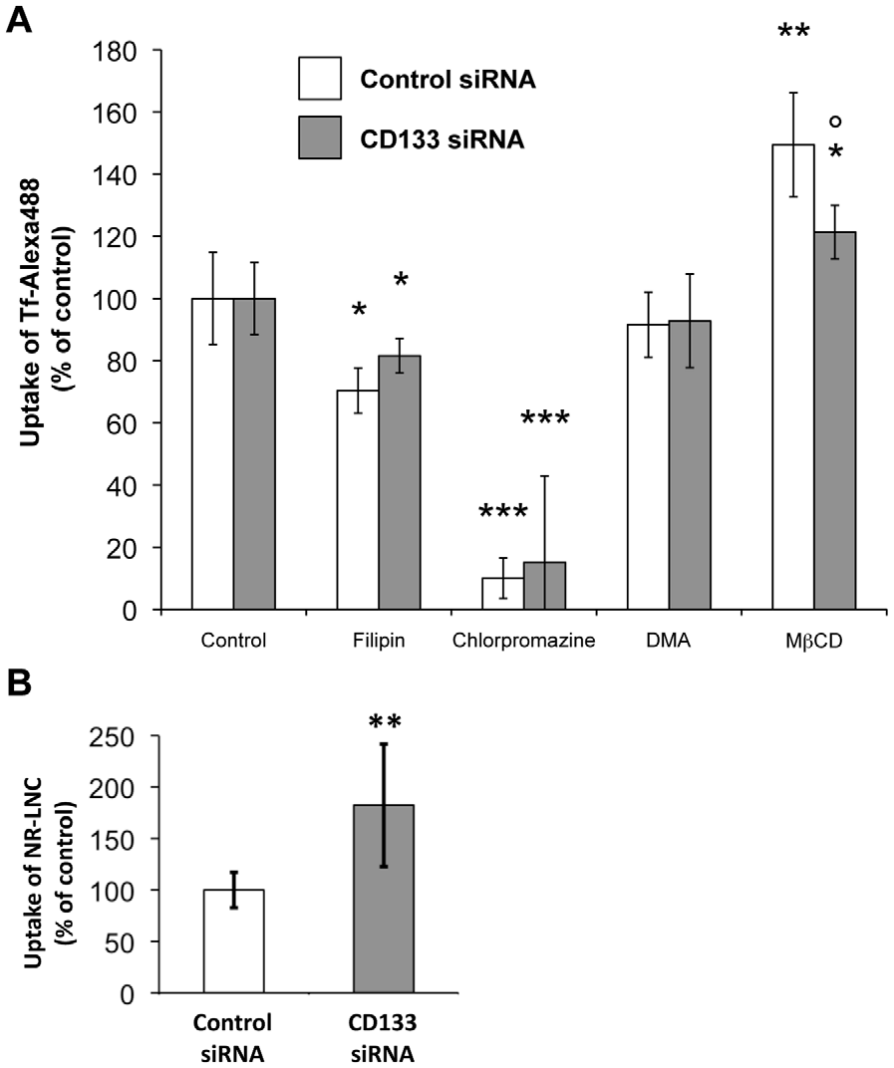
Implication of CD133 in endocytosis mainly involved the clathrin pathway. A) Consequences of CD133-specific siRNA knockdown for the effectiveness of chemical modulators of known endocytic pathways in modulating Tf uptake by non-differentiated Caco-2 cells. After treatment with either vehicle alone (control), filipin, chlorpromazine, DMA or MβCD added with lovastatin (MβCD), CD133^high^ (control siRNA) and CD133^low^ (CD133 siRNA) non-differentiated Caco-2 cells were exposed to 5 µg/mL Tf-Alexa 488. Cellular internalization of Tf-Alexa 488 was then monitored by flow cytometry. Results are expressed as a % of Tf-Alexa 488 amounts that were internalized in the vehicle treated control. Note the absence of effect of CD133-siRNA knockdown on the major inhibition of Tf-uptake caused by chlorpromazine. Note also the reduced up regulatory effect of cholesterol extraction in the CD133 low situation (MβCD). Data represented mean ± s.e.m. of a triplicate obtained from one representative experiment that was reproduced twice. Comparisons with control: Dunnett's test, *p<0.05, **p<0.01, ***p<0.001; comparison between control siRNA and CD133 siRNA: Dunnett's test: p<0.05. B) Specific siRNA mediated knockdown of CD133 within non-differentiated Caco-2 cells led to an increase in LNC intracellular accumulation. Flow cytometric analysis of intracellular uptake of NR-LNC within CD133^high^ (Control siRNA) and CD133^low^ (CD133 siRNA) Caco-2 cells after 1 h of incubation at 37°C/5%CO_2_. Results are expressed as percentage of control, thus representing the geomean fluorescence intensity levels obtained for cells treated with vehicle alone. Data represented mean ± s.e.m. obtained from three independent experiments. Dunnett's test: **p<0.01.

Having established that the level of expression of CD133 had an impact on Tf uptake within non-differentiated Caco-2 cells but did not modulate Tf recycling, we then addressed the question whether CD133 is involved in specific pinocytic pathways [33]. For this purpose, uptake of Tf-Alexa 488 within CD133^high^ and CD133^low^ expressing cells was monitored after treatment with previously identified chemical inhibitors of known endocytic pathways. Chlorpromazine was used to inhibit clathrin mediated transport [22], filipin to inhibit caveolae dependent endocytosis [23] and DMA to inhibit macropinocytosis [24]. Flow cytometry established that pretreatment of control CD133^high^ Caco-2 cells with filipin, chlorpromazine and DMA before exposure to 5 µg/mL Tf-Alexa 488, led to a 30%, 90% and non-significant reduction in Tf uptake, respectively (Figure 3A). The major impact of chlorpromazine combined with the slighter effect of filipin thus emphasized that Tf accumulation within non-differentiated Caco-2 cells was mainly due to clathrin-mediated transport [37]. The filipin effect could be explained by the fact endocytosis of protopic substrates for receptor-mediated endocytosis, such as LDL or Tf, which usually route to the intracellular compartment through the clathrin pathway, could alternatively use the caveolae pathway [38], [39]. Moreover, as cholesterol has been shown to be involved in the formation of clathrin coated endocytic vesicles [26], filipin, which sequestrates cholesterol, may also have an indirect impact on clathrin endocytosis. Interestingly, when considering CD133 knockdown and CD133^low^ Caco-2 cells, very similar data were obtained with filipin, chlorpromazine and DMA, leading to 18%, 85% and non-significant reduction in Tf uptake, respectively (Figure 3A). Thus, the quantitative effects of CD133 on Tf endocytosis appear to be mainly related to the clathrin-dependent pathway. Surprisingly, cholesterol depletion, achieved by using MβCD combined with lovastatin [25], [26], which has been said to affect clathrin independent pathways and to some extent clathrin dependent pathways [26], [40], [41], resulted in a major improvement in Tf-Alexa 488 accumulation within CD133^high^ Caco-2 cells (+49%, Figure 3A). Interestingly, the impact of cholesterol depletion on Tf-Alexa 488 uptake within CD133^low^ Caco-2 cells was less marked (+21%, Figure 3A). These last data supported the hypothesis that CD133 interaction with intracellular cholesterol also has an impact on Tf endocytosis. To further corroborate a possible link between CD133 and the clathrin-dependent endocytosis pathway, impact of CD133 knockdown on the uptake of synthetic nanoparticles (LNC) that were previously described to use the clathrin pathway on route to the intracellular space of Caco-2 cells [21] was investigated. Interestingly, as for fluorescent Tf, uptake of fluorescently tagged LNC (NR-LNC) was significantly higher in CD133^low^ Caco-2 cells (Control siRNA) as compared with CD133^high^ Caco-2 cells (CD133 siRNA) (Figure 3B).

### Treatment of non-differentiated Caco-2 cells with the AC133 antibody recognizing the extracellular domain of CD133 resulted in a reduction in Tf uptake

**Figure 4:**
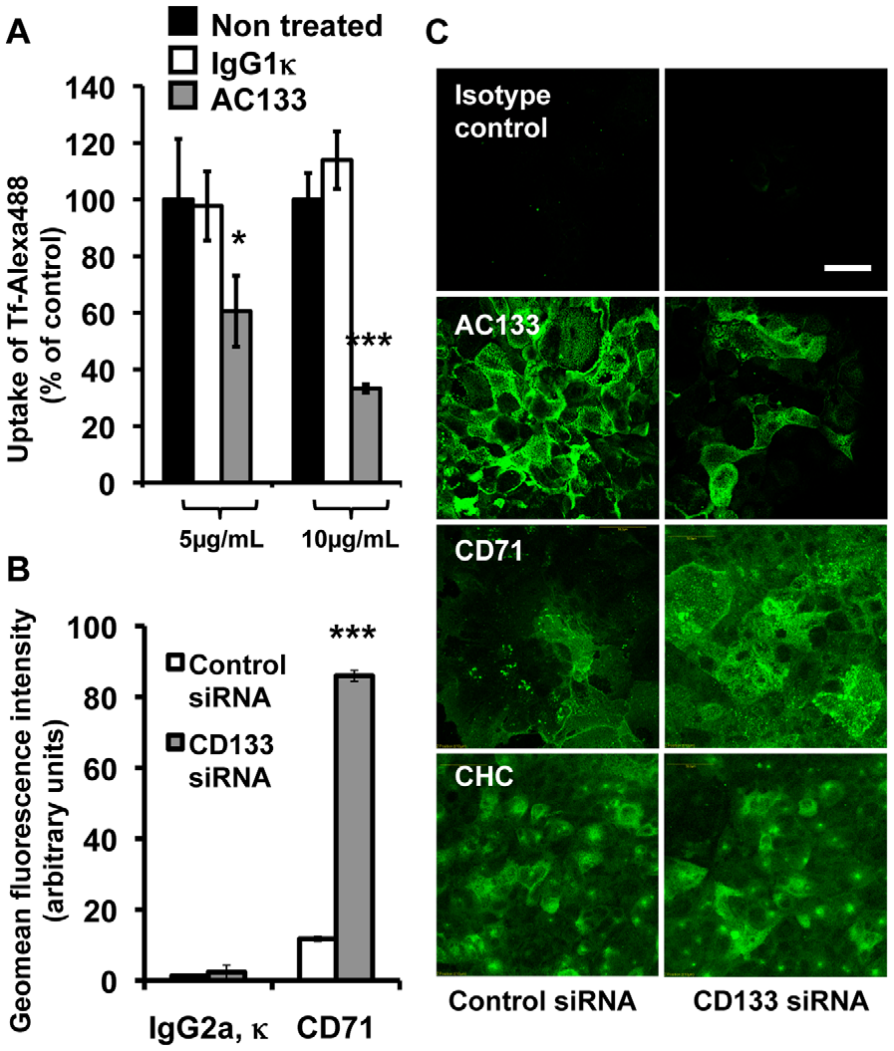
Recognition of CD133 and modulation of its expression interfere with Tf uptake and TfR accessibility in Caco-2 cells. A) AC133 antibody treatment inhibited Tf uptake. Constitutively CD133-expressing undifferentiated Caco-2 cells were exposed to Tf-Alexa 488 for 1 h at 37°C/5%CO_2_ in the presence of 5 or 10 µg/ml of AC133 or IgG1κ immunoglobulin control. Tf-Alexa 488 that was effectively internalized within cells was then monitored by flow cytometry. Results are expressed as a % of Tf-Alexa 488 amounts that were internalized in the untreated control. Data represented mean ± s.e.m. from a triplicate obtained from one representative experiment. Dunnett's test: *p<0.05, ***p<0.001. B) Flow cytometric analysis of the expression of TfR (CD71) at the surface of CD133^high^ (Control siRNA) and CD133^low^ (CD133 siRNA) Caco-2 cells. Data represented mean ± s.e.m. obtained from three independent experiments. IgG2aκ immunoglobulins were used as negative control immunostaining. Dunnett's test: ***p<0.001. C) Analysis of the expression of AC133, TfR/CD71 and CHC within Caco-2 cells by immunocytochemistry combined with confocal laser scanning microscopy. Note the increase of CD71 expression while AC133 was depleted from the Control siRNA to the CD133 siRNA situation. Bar: 50 µm.

Having established that CD133 expression quantitatively affected Tf endocytosis, we then considered that a direct interaction between the CD133 protein and the Tf endocytic machinery would have been affected by extracellular ligand binding to CD133. As extracellular ligand of CD133 had not yet been identified and since monoclonal antibodies have already been used as activating or as function blocking immunoglobulins, for instance in interactions between integrins and extracellular matrix [42], the AC133 antibody, which recognizes an extracellular glycosylation associated epitope of CD133 [43], was then tested as CD133 ligand on living Caco-2 cells. Interestingly, while treatment of non-differentiated Caco-2 cells with an IgG1κ isotype control immunoglobulin had no effect on the intracellular accumulation of Tf-Alexa 488 evaluated by flow cytometry (Figure 4A), treatment with the AC133 antibody resulted in a major reduction in Tf uptake of 38±8% and 75±1% at 5 and 10 µg/mL, respectively (Figure 4A). These data established that AC133 can effectively exert functional effects on living cells here ascribed to an interaction between CD133 itself and the Tf endocytic machinery in non-differentiated Caco-2 cells.

### siRNA knowkdown of CD133 resulted in a strong improvement of the TfR accessibility from the extracellular space

To further assess the accessibility to the TfR (or CD71) from the extracellular compartment depending on the presence of CD133, expression of the TfR/CD71 at the plasma membrane was evaluated on CD133^low^ Caco-2 cells (Control siRNA) and CD133^high^ Caco-2 cells (CD133 siRNA). Immunofluorescence combined with flow cytometry analysis demonstrated a dramatic improvement of CD71/TfR recognition by the corresponding antibody when CD133 was concomitantly down regulated through siRNA knockdown (from 11.74 to 85.94 arbitrary units, p<0.001, Figure 4B). Immunocytochemistry combined with confocal laser scanning microscopy confirmed this observation (Figure 4C). Combined with the direct short-term effect of the AC133 antibody on the uptake of Tf, these data supported a direct or indirect interaction of CD133 with CD71/TfR at the plasma membrane. In contrast, although the clathrin pathway was found to be the major endocytic process for Tf within Caco-2 cells, no evident differences were detected by immunocytochemistry in the pattern of expression of CHC (Figure 4C).

### Relation between iron supplementation or deprivation and AC133/CD133 expression

**Figure 5:**
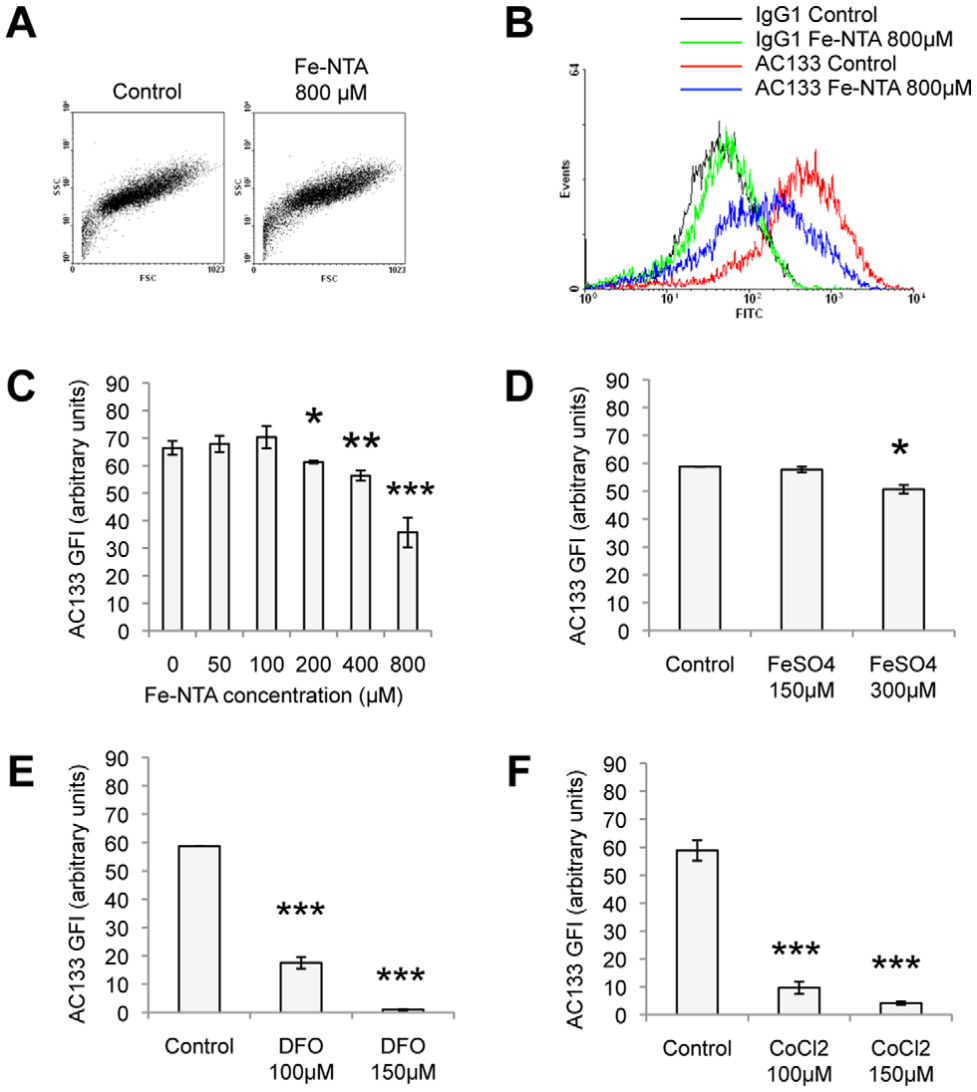
Iron supplementation down regulated AC133/CD133 expression in Caco-2 cells in a dose-dependent fashion. Combined immunofluorescence and flow cytometry was used to assess the impact of Fe-NTA treatment on AC133/CD133 expression in Caco-2 cells. A) Forward scatter (FSC, approximate cell size) and side scatter (SSC, cell complexity or granularity) profiles of vehicle only treated cells (control) versus Fe-NTA (800 µM) treated cells. B) Cell number distribution versus FITC-fluorescence (arbitrary units) in control and Fe-NTA (800 µM) treatments after IgG1 control or AC133 immunostaining. Dose-response histograms representing AC133/CD133 expression as a function of Fe-NTA (C), FeSO_4_ (D), DFO (E) or CoCl_2_ (F) treatments at different concentrations as indicated. GFI: geomean fluorescence intensity. Data represent mean ± s.e.m. of a triplicate obtained from one representative experiment. Dunnett's test: *p<0.05, **p<0.01, ***p<0.001.

Although Tf-independent iron transport can occur in mammalian cells [44], the majority of extracellular iron is bound to Tf [45] and uptake of iron occurs from iron loaded Tf through TfR dependent endocytosis [33]. Interestingly, most of the proteins involved in iron metabolism are regulated by iron itself notably through iron regulatory proteins (IRP-1, IRP-2) that, in iron starved cells, bind to cis-acting elements called iron responsive elements (IREs) located in the 5′ or 3′UTR of transcripts [46], [47], [48]. Thus, binding of IRPs to IREs located in the 5′UTR of targeted messengers leads to inhibition of translation, as exemplified by ferritin, which is up regulated in response to high iron concentrations [49]. In contrast, recognition of IREs located in the 3′UTR of transcripts lead to their stabilization, as exemplified by Tf, which is down regulated in response to high iron concentrations [50]. Since the present work established that AC133/CD133 regulated endocytosis of holo-Tf in Caco-2 cells, we further addressed the question whether AC133/CD133 was in turn regulated by iron. For this reason, non-differentiated Caco-2 cells, previously placed in serum free medium, were treated with extemporaneously prepared Fe-NTA (1∶4) for 72 h at 37°C/5% CO_2_ as described by others [44], [51], [52], thus allowing cells to improve their iron contents. The impact of Fe-NTA treatment on CD133/AC133 expression was then assessed by flow cytometry (Figure 5A–C). As exposure to extracellular iron has previously been shown to be toxic for cells [53], we checked that Fe-NTA concentrations ranging from 50 to 800 µM had no impact on cell shape and attachment (data not shown) or on cell death (Figure 5A). Interestingly, while low concentrations of Fe-NTA did not alter CD133/AC133 expression, 200 to 800 µM Fe-NTA induced dose dependent down regulation of cancer stem cell marker expression (Figure 5B–C). To confirm that iron supplementation regulated CD133/AC133 expression, Caco-2 cells were treated with FeSO_4_, another iron donor [27], [28]. As shown on Figure 5D, FeSO_4_ at 300 µM lead to a down regulation of CD133/AC133 expression, while having no effect at 150 µM. To further understand the modality of regulation of CD133/AC133 expression by iron, iron chelation with DFO was also investigated. Interestingly, DFO treatment significantly reduced AC133 expression in Caco-2 cells in a dose dependent manner at 100 and 150 µM (Figure 5E). As iron supplementation expectedly had opposite effect as iron deprivation on intracellular iron content, the reduction of AC133 expression in both situations suggests the possibility that supplementation and deprivation may work on distinct pathways. As such, DFO is also known as a hypoxia-mimetic agent, which by inhibiting the activity of prolyl hydroxylases stabilizes the HIF-1α transcription factor [29], [30]. To further cope with the hypothesis that DFO-induced hypoxia may down regulate the expression of AC133/CD133, effect of CoCl_2_ [31], a hypoxia-mimetic agent that stabilize HIF-1α and works independently from iron deprivation, was investigated. Figure 5F established that, similarly to DFO, CoCl_2_ at 100 and 150 µM strongly inhibited the expression of AC133/CD133 within Caco-2 cells. This observation emphasized that control of AC133/CD133 expression by iron in Caco-2 cells may in parallel to mRNA stabilization, also involved transcriptional regulation notably through HIF-1α.

**Figure 6:**
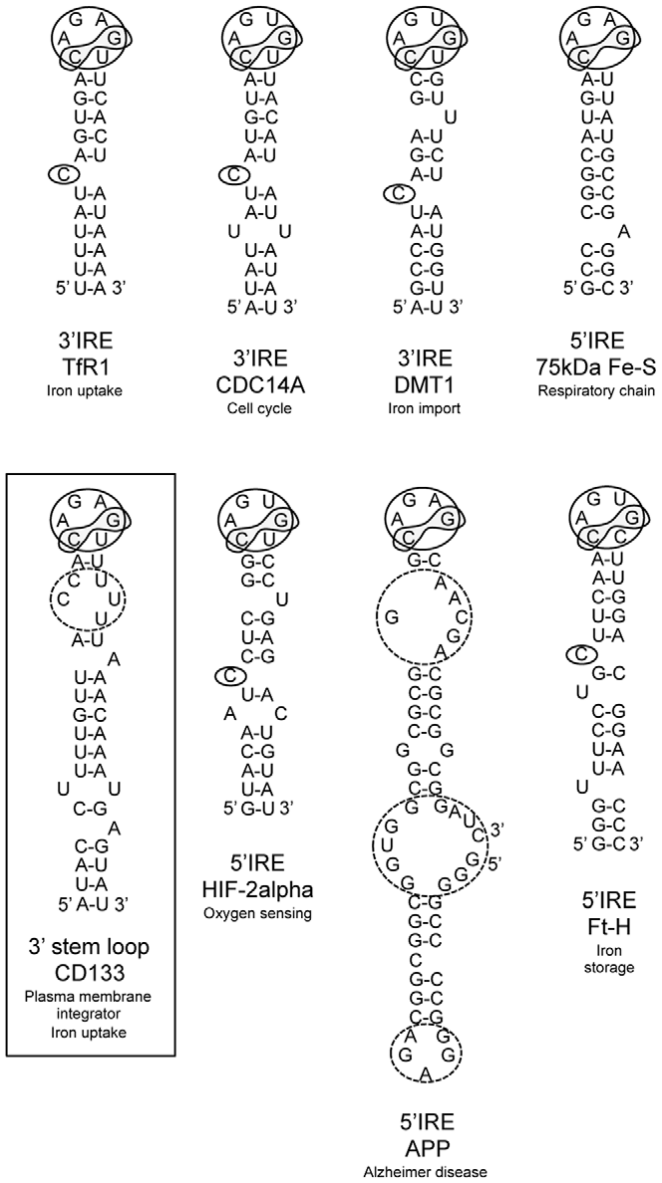
Schematic representation of known IREs and nearest related stem loop sequence located in the 3′UTR of the CD133 mRNA (NCBI GenBank accession number NM_001145847.1, nucleotide sequence: from +3544 to +3574). Note the perfect match between the 5′-ACAGAGUU-3′ loop sequence of the CD133 mRNA and the one present in the TfR1 3′IRE. Note also the high discrepancy between hairpin structures, notably with the presence of secondary loops (dashed line) in the APP 5′IRE. A secondary loop is also present in the CD133 hairpin selected here. CDC14A: dual specificity protein tyrosine phosphatase. DMT1: divalent metal transporter 1. 75 kDa Fe-S: NADH dehydrogenase (ubiquinone) Fe-S protein 1. APP: Alzheimer amyloid precursor protein. Ft-H: ferritin heavy chain.

To get more information on the direct or indirect effect of iron on AC133/CD133 expression, a search for putative IRE-like stem loops within the 5′UTR and 3′UTR of human CD133 mRNA was carried out using computer based sequence alignments as described in Materials and Methods. Although previously published IRE sequences [32], [47], [48], [54], [55], [56], [57], [58] were not recognized, and the SIREs Web research [32] failed to detect any classical stem loop sequence within the 5′UTR and 3′UTR of human CD133 mRNA, a hairpin loop presenting the non-canonical CAGAGU sequence as observed in the first IRE identified in the human TfR mRNA was identified in the 3′UTR. This selected sequence, which still requires investigation, is presented among validated IREs in Figure 6.

## Discussion

Our results provide evidence for an important role of the 5-domain trans-membrane molecule CD133 in the regulation of cellular cross-talk with the extracellular microenvironment, revealed firstly by inhibition of Tf endocytosis in non-differentiated Caco-2 cells, secondly by the ability of the CD133-specific AC133 antibody to also inhibit Tf endocytosis and thirdly by the negative regulation of the TfR accessibility. Since Tf is the main iron carrier entry to the mammalian cell, the identification of a role of CD133 in endocytosis must be related to the regulation of CD133 itself by iron, thus supporting a possible Tf-CD133-iron network in cell metabolism.

### CD133 as an endocytosis modulator

The unique localization of the cholesterol binding protein CD133 in plasma membrane protrusions and microvilli, as well as in association with specific lipid raft microdomains that are sensitive to Triton X100 but resistant to Lubrol WX supported the hypothesis that CD133 has a significant function in plasma membrane organization [14], [59], [60]. The role of CD133 was suggested by the generation of apical membrane protrusions from epithelial cells and notably in the release of extracellular membrane particles, which themselves carry CD133 [16], [61], [62]. By further establishing, to our knowledge for the first time, a role for CD133 in endocytosis, importantly the present study extends the functionality of CD133 at the plasma membrane interface. Thus, the quantitative effects of CD133 on Tf endocytosis, as well as on LNC, can be mainly ascribed to the clathrin pathway, which is the main Tf-internalization pathway within Caco-2 cells [33]. This assertion was also corroborated by the absence of effect of CD133 knockdown on the uptake of Dx and CTB that are usually internalized through fluid phase and caveolae dependent endocytosis, respectively [33]. Hence, CD133-dependent up regulation of Tf uptake after cholesterol extraction (MβCD treatment) supported the hypothesis that CD133, at least partially, inhibits Tf endocytosis via a cholesterol dependent mechanism. Accordingly, cholesterol extraction either up regulates Tf uptake via an unknown compensatory mechanism or alternatively, reduces constitutive extracellular Tf recycling. It has previously been established that acute cholesterol extraction can increase the number of TfR on the cell surface [41], [63], thus explaining relatively higher Tf entry to the cell, notably in the inhibitory CD133^high^ situation. Also, absence of a differential effect of filipin treatment depending on CD133 expression emphasized the fact that cholesterol extraction (MβCD treatment) has different effects than cholesterol sequestration (filipin treatment) on pinocytic pathways. However, since the influence of CD133 on Tf endocytosis involved the clathrin pathway and cellular cholesterol, an interaction of the CD133 molecule with the TfR, whatever the endocytic pathway involved, cannot be excluded. Hence, the effect of the AC133 antibody on Tf endocytosis may either support steric shielding affecting the binding of Tf to its receptor or, instead, signaling through the CD133 molecule that affects Tf-TfR uptake. The dramatic up regulation of the TfR expression at the plasma membrane of Caco-2 cells when CD133 was concomitantly down regulated validated such interaction between CD133 and the TfR. Among signaling pathways, a previous work demonstrated CD133 phosphorylation of the cytoplasmic domain by Src and Fyn tyrosine kinases [5]. Whether the presence of the AC133 epitope in the CD133 extracellular domain and binding to an as yet unidentified ligand influences CD133 phosphorylation and resulting signaling remains a major question. CD133 may thus modulate Tf uptake through direct inhibitory effects on TfR or on the structure of TfR microdomains. Moreover, alteration of ongoing mechanisms that promote Tf-TfR turnover may be involved: for instance, insulin stimulated redistribution of TfR to the plasma membrane [64], hemochromatosis protein co-trafficked with the TfR to the cell surface [65] and PI3kinase-mTOR regulated the number of TfR per endocytic vesicle [66]. Alternatively, as CD133 is a glycosylated molecule, glycosylation could be important for the regulation of Tf binding to the cell surface and likely in iron metabolism [67].

### CD133 as an integrator of cell metabolism

Supporting the aerobic glycolysis of cancer cells first reported by Otto Warburg in the 1950s [68], it was recently established that CD133 expression is associated with high cellular glucose metabolism: CD133 was found to be a glucose responsive gene in L6 myotubes [11] and CD133 expression to be concomitant to high glucose cellular uptake in U251 glioma cells [12]. Since co-localization of glucose transporters with the TfR in intracellular vesicles has previously been observed [69], [70], [71], whether CD133 can regulate iron uptake together with glucose transport by modulation of endocytosis remains an important question. The combined sequestration of TfR and glucose transporter at the plasma membrane would be expected to result in low iron levels combined with high glucose intracellular levels. As iron-sulfur cluster assembly [72] is an important process for mitochondria activity, inhibition of a respiratory phenotype may well fit with the appearance of a glycolytic one [73]. Having established here that CD133 knockdown up regulated holo-Tf uptake and that iron supplementation and deprivation down regulated CD133 expression, a role for CD133 as an integrator of iron metabolism, with CD133 and TfR being jointly modulated [48], is suspected. In line with this hypothesis, direct effects on putative IREs may be involved in the regulation of CD133 expression by iron. Considering homology but also a degree of variability among IREs, as for instance between TfR 3′IRE [48] and APP 5′IRE [58] (Figure 6), the stem loop sequence we selected for possible CD133 3′IRE (Figure 6) remained to be either validated or rejected. Alternatively, indirect regulation of CD133 expression may be implicated. As an illustration of the latter, also supported by our experiments using DFO and CoCl_2_, oxygen sensing [74], [75] and mTOR [76] have been shown to regulate CD133 expression via activation of the HIF-1α transcription factor. Since iron treatment can in turn modulate HIF-1α activity [77], [78], [79], iron induced destabilization of HIF-1α may also explain down regulation of CD133 expression in non-hypoxic conditions [80].

### Consequences for cancer cell behavior and for detoxification status

It has previously been established that the AC133 epitope is lost during Caco-2 cell differentiation but not the CD133 protein [60]. In addition, differentiation of Caco-2 cells up regulates genes implicated in iron transport and metabolism, including ferroportin, Tf and TfR [81]. Thus, whether causative or not, improved iron metabolism appears to be correlated with the differentiation status of Caco-2 cells. Whether iron regulation of AC133 expression is a basic signal for differentiation or/and for cancer cell recruitment to a secondary phenotype is of pivotal interest. Also, several extrinsic signaling molecules have already been linked to the loss of expression of AC133 associated with differentiation, notably in brain tumor stem cells with BMP-4 [82], retinoic acid [83] or oxygen [74], [75]. Our identification of a role for CD133/AC133 in inhibiting endocytosis points to another consequence of cellular cross-talk between cancer cells and their microenvironment, or of cancer stem cells and the stem cell niche, that is a finest control of extrinsic signals. Indeed, corruption of the niche as well as the exclusion of toxic metabolites and xenobiotics appears to be an important mechanism to define the status of cancer stem cells, with the occurrence of side population cells that express specific efflux transporters [84]. Hence, a fine control of iron accumulation might prevent the generation of deleterious reactive oxygen species via iron catalyzed Fenton chemistry [46], with a possibly impact on iron-induced carcinogenesis [85] and on degenerative diseases [86], [87]. Since a major phenotype for CD133 loss was disk dysmorphogenesis and photoreceptor degeneration [7] and since iron toxicity can trigger retinal degeneration [88], the present work emphasized the need for further exploration of the newly revealed CD133-Tf-iron network.
